# A review of “music and movement” therapies for children with autism: embodied interventions for multisystem development

**DOI:** 10.3389/fnint.2013.00022

**Published:** 2013-04-09

**Authors:** Sudha M. Srinivasan, Anjana N. Bhat

**Affiliations:** ^1^Department of Kinesiology, Neag School of Education, University of ConnecticutStorrs, CT, USA; ^2^Center for Health, Intervention, and Prevention, University of ConnecticutStorrs, CT, USA; ^3^Center for the Ecological Study of Perception and Action, University of ConnecticutStorrs, CT, USA

**Keywords:** music, movement, motor, social, communication, autism, children

## Abstract

The rising incidence of Autism Spectrum Disorders (ASDs) has led to a surge in the number of children needing autism interventions. This paper is a call to clinicians to diversify autism interventions and to promote the use of embodied music-based approaches to facilitate multisystem development. Approximately 12% of all autism interventions and 45% of all alternative treatment strategies in schools involve music-based activities. Musical training impacts various forms of development including communication, social-emotional, and motor development in children with ASDs and other developmental disorders as well as typically developing children. In this review, we will highlight the multisystem impairments of ASDs, explain why music and movement therapies are a powerful clinical tool, as well as describe mechanisms and offer evidence in support of music therapies for children with ASDs. We will support our claims by reviewing results from brain imaging studies reporting on music therapy effects in children with autism. We will also discuss the critical elements and the different types of music therapy approaches commonly used in pediatric neurological populations including autism. We provide strong arguments for the use of music and movement interventions as a multisystem treatment tool for children with ASDs. Finally, we also make recommendations for assessment and treatment of children with ASDs, and provide directions for future research.

## Introduction

Autism Spectrum Disorders (ASDs) are a group of neurological disorders characterized by social communication impairments as well as the presence of stereotyped and repetitive behaviors and interests (American Psychiatric Association, 2000). Children with ASDs demonstrate social impairments such as poor social and emotional reciprocity or turn taking and reduced eye contact during social exchanges (Mundy and Crowson, 1997; Dawson et al., 2004). Communication impairments in autism typically involve the lack of or a delay in the acquisition of language, difficulties in initiating and sustaining conversations with social partners, and the idiosyncratic use of language (Tager-Flusberg, 1999). In addition, the presence of repetitive and stereotypical behaviors is a hallmark of autism; children with ASDs demonstrate repetitive manipulations of objects, stereotypical behaviors such as flapping of hands, twisting of the body, and compulsive behaviors such as inflexible adherence to fixed routines and rituals (Bodfish et al., 2000; Boyd et al., 2012). In addition to these core impairments, children with ASDs may demonstrate several secondary impairments or comorbidities including significant behavioral and emotional problems as well as perceptuo-motor impairments. Behavioral and emotional problems include anxiety, aggression, depression, hyperactivity, temper tantrums, and/or self-injurious behaviors (Bodfish et al., 2000; Lecavalier, 2006; Loh et al., 2007; Mazefsky et al., 2012). A growing body of evidence suggests that perceptuo-motor impairments are frequently present in children with ASDs (Fournier et al., 2010; Bhat et al., 2011). Specifically, children with autism have difficulty modulating sensory inputs (Baranek, 1999; Baranek et al., 2005; Tomchek and Dunn, 2007) which may manifest as enhanced perception of auditory and visual stimuli (Bonnel et al., 2003; Heaton, 2003; Gernsbacher et al., 2008). Furthermore, they have significant and pervasive motor impairments such as problems with dual and multi-limb coordination (Green et al., 2009; Fournier et al., 2010), postural control (Minshew et al., 2004), gait (Vilensky et al., 1981; Hallett et al., 1993), as well as imitation and praxis (Mostofsky et al., 2006; Dewey et al., 2007). Comorbidities in perceptuo-motor performance could contribute to the social communication impairments of ASDs. Specifically, limited movement exploration and motor clumsiness may lead to missed opportunities to develop social connections with peers and caregivers (Leary and Hill, 1996; Jansiewicz et al., 2006; Bhat et al., 2011). Taken together, ASDs are multisystem disorders with both primary social communication impairments and secondary perceptuo-motor and behavioral comorbidities.

The current standard of care for ASDs includes the use of Applied Behavioral Analysis (ABA) (Lovaas, 1987), Picture Exchange Communication Systems (PECS) (Bondy and Frost, 2003), Teaching and Education of Autistic and Related Communication Handicapped Children (TEACHH) (Mesibov et al., 2004) as well as developmental, skill-based approaches (Pierce and Schreibman, 1995; Kasari et al., 2008). ABA, PECS, and TEACHH approaches recommend specific strategies for social interaction and environmental structure to promote positive behaviors and communication in children with ASDs (Lovaas, 1987; Bondy and Frost, 2003; Mesibov et al., 2004). The developmental approaches promote specific early social communication skills such as joint attention and imitation. While these approaches have significant evidence to support their use, they are primarily used to promote social communication and academic skills (Landa, 2007). Few approaches such as Sensory Integration therapy (Baranek, 2002) or Floortime (Greenspan and Wieder, 1999) promote perceptuo-motor development; however, there is limited evidence to support their use. Given the multisystem nature of the impairments in ASDs, there is a clear need to develop multisystem interventions that address their core social communication deficits as well as their perceptuo-motor and behavioral comorbidities. In this review, we highlight the multisystem effects of music therapies and how they might benefit children with ASDs.

Music-based therapies form about 12% of all autism interventions and 45% of all alternate treatment strategies used within school settings (Simpson et al., 2005; Hess et al., 2008). However, our review of published and unpublished research evaluating the efficacy of music therapies in autism revealed that the majority of the studies involved single-subject designs or small sample sizes (see Table 1). Moreover, these studies involved a pre-post comparison of outcomes in the treatment group and did not include a control group. The overall quality of studies was poor except for three published randomized controlled trials (Lundqvist et al., 2009; Lim, 2010; Gattino et al., 2011). The majority of the studies focused on addressing the communication impairments in autism. Few studies used musical experiences to facilitate social-emotional and behavioral outcomes in ASDs (see Table 1 for details). Interestingly, the effects of music therapy on motor performance and motor stereotypies have never been examined. Given the current state of the music therapy literature, it is difficult to make definitive claims about the effects of music-based interventions in children with ASDs, except for the significant treatment effects in improving communication. In this review, we not only acknowledge the limitations of the music therapy literature, but also provide additional sources of evidence from the fields of music education, neuroscience, and special education to make a strong case for “music and movement” activities as multisystem interventions for children with ASDs. We believe that the multisystem nature of musical experiences warrants further systematic investigation as an effective treatment strategy to address both the core impairments and comorbidities of individuals with autism.

**Table 1:** **Music therapies in children with Autism Spectrum Disorders (ASDs)**.

**Study**	**Sample size**	**Age of subjects in years**	**Therapy duration (number of days)**	**Therapy frequency (number of sessions per week)**	**Type of intervention (Active/Passive)**	**Type of music used (Live/Recorded)**	**Intervention design (Individual/Group)**
**STUDIES ASSESSING COMMUNICATION OUTCOMES**							
Gattino et al., 2011	24	6.7–12.2	16	1	Active	Live	Individual
Wan et al., 2011	6	5.9–8.9	40	5	Active	Live	Individual
Lim, 2010	51	3–5	6	3	Passive	Recorded	Individual
Edgerton, 1994	11	6–9	10	1	Active	Live	Individual
Buday, 1995	10	4.4–9	8	4	Passive	Recorded	Individual
Lim and Draper, 2011	22	3–5	3	6	Active	Live	Individual
Corbett et al., 2008	11	3–7	38	7	Passive	Recorded	Individual
**STUDIES ASSESSING SOCIAL OUTCOMES**							
Kim et al., 2008	15	3–5	12	1	Active	Live	Individual
**STUDIES ASSESSING EMOTIONAL OUTCOMES**							
Katagiri, 2009	12	9–15	8	2	Active and Passive	Live and Recorded	Individual
Kim et al., 2009	15	3–5	12	1	Active	Live	Individual
**STUDIES ASSESSING BEHAVIORAL OUTCOMES**							
Lundqvist et al., 2009	20	22–57	10	2	Passive	Recorded	Individual
Boso et al., 2007	8	23–38	52	1	Active	Live	Group
Carnahan et al., 2009a,b	6	6–11	40	5	Active	Recorded	Group

*Note: This table does not include case studies or unpublished theses and dissertations*.

We propose that music-based interventions are effective treatment tools for individuals with ASDs because they harness the musical strengths of this population while alleviating their impairments. We are offering three different reasons that make music-based interventions particularly attractive for individuals with ASDs. First, musical training may help address the various core autism impairments in joint attention, social reciprocity, and non-verbal and verbal communication, as well as comorbidities of atypical multisensory perception, poor motor performance, and behavioral problems. Second, children with ASDs find musical activities enjoyable, perhaps due to their enhanced musical understanding (Heaton, 2003). Children with autism have enhanced pitch perception abilities compared to typically developing children, for instance, enhanced pitch memory, labeling (Heaton, 2003), and discrimination (Bonnel et al., 2003). Therefore, clinicians and special educators often use music-based activities in school settings to engage children with ASDs (Hess et al., 2008). Third, music-based activities can be non-intimidating experiences wherein a child with ASD spontaneously explores various musical instruments, with the trainer joining in and copying the child's actions. Children with ASDs have difficulties with direct social engagement; hence, socially embedded group musical activities provide excellent opportunities to engage in predictable and comfortable interactions with social partners (Darrow and Armstrong, 1999; Allgood, 2003). In this review, we first provide evidence for the multisystem effects of musical experiences in facilitating various skills in children with autism, other neurological populations, and healthy individuals. Next, we discuss the critical elements of music-based activities and the popular music therapy approaches used in ASDs and other pediatric developmental disorders. Finally, based on the current literature, we provide recommendations for clinicians and clinical researchers working with children with autism including ideas for assessment and treatment.

## Multisystem effects of musical experiences

**Figure 1:**
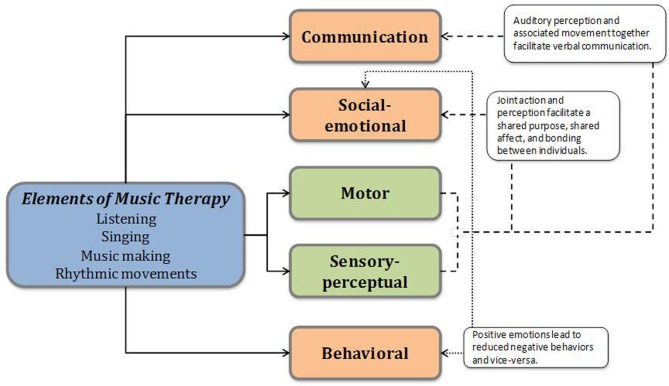
**Direct and indirect influences of musical experiences/therapies on the various domains of development**.

In this section, we describe the supporting evidence for how embodied music therapies promote communication, social-emotional, perceptuo-motor, and behavioral skills in children with ASDs. In each sub-section, we will first explain the mechanism for positive effects of musical experiences and the evidence supporting the use of embodied music interventions in remediating the impairments in autism. Since the current research on music-based therapies in autism is limited, we will also rely on evidence from healthy individuals and pediatric populations with similar neurological impairments as autism. Figure 1 shows the direct and indirect effects of musical experiences on the perceptuo-motor, communication, social-emotional, and behavioral domains of development. We will also offer recent neuroscientific evidence which suggests that musical experiences may shape the nervous system in healthy individuals and discuss its implications for individuals with ASDs.

### Effect of musical experiences on the development of language and communication

Musical experiences involving singing, chanting, and playing of musical instruments clearly require communication between individuals. Music and language are closely related to each other in that both music and language are hierarchically arranged, with lower-level units such as notes/keys or letters/syllables integrated to form higher-level units such as chords/chord progressions or words/sentences (Molnar-Szakacs and Overy, 2006). Moreover, music and language are strikingly similar in the complexity of acoustic information, the use of spatial notation such as musical notation and the alphabet (Kraus and Chandrasekaran, 2010), as well as cognitive processes such as attention and memory (Patel et al., 1998; Foxton et al., 2003). These similarities allow easy transfer of learning between music and language (Tallal and Gaab, 2006). Children with ASDs have significant communication impairments despite relatively preserved musical skills (Bonnel et al., 2003; Heaton, 2003). Hence, music therapies have been used to facilitate verbal and gestural communication skills in children with ASDs (Edgerton, 1994; Buday, 1995; O'Loughlin, 2000; Farmer, 2003; Gold et al., 2006; Lim, 2010; Tindell, 2010; Gattino et al., 2011; Lim and Draper, 2011; Simpson and Keen, 2011; Wan et al., 2011) (see Table 1). A recent meta-analysis revealed that active music therapies involving singing and music-making led to significant improvements in verbal communication skills and non-verbal, gestural communication skills in children with ASDs (Gold et al., 2006). Effect sizes varied between 0.4 and 0.5 based on two randomized control trials involving 20 participants in the music therapy group compared to the control “placebo” therapy group (Buday, 1995; Farmer, 2003; Gold et al., 2006). Overall, there is some evidence from the autism literature supporting the links between music and language, thus justifying the use of music therapies to enhance communication skills in autism.

Literature from music education suggests strong links between musical training and enhanced communication skills in typically developing children and adults. Prolonged music training not only enhances musical perception but also speech perception/receptive language as well as expressive language (Butzlaff, 2000; Jakobson et al., 2003; Schlaug et al., 2005; Magne et al., 2006; Forgeard, 2008; Kraus and Chandrasekaran, 2010). Children and adults who received long-term musical training showed significant advances in basic auditory perception of music as well as speech, particularly, pitch perception (Schön et al., 2004; Marques et al., 2007; Moreno et al., 2009). Adult musicians were better able to detect weak violations/incongruities in pitch within both music and language compared to non-musicians (Schön et al., 2004). Moreover, the ability to detect pitch violations in language was not restricted to their native language; it also generalized to foreign languages (Marques et al., 2007). Similar enhancements in pitch perception were observed in children who had at least 4 years of musical training (Magne et al., 2006). Even children who received short-term musical training for a 6-month period were better able to detect weak pitch violations in both music and speech than children who received painting training (Moreno et al., 2009). Other perceptual skills that improve with prolonged musical training include rhythmic and auditory discrimination abilities (Jakobson et al., 2003) as well as melodic contour perception (Forgeard, 2008). Musical training not only enhances music and speech perception but also directly impacts expressive language. Musically trained children outperformed musically naïve children on tasks of verbal memory, verbal fluency, and non-verbal reasoning (Ho et al., 2003; Forgeard, 2008).

Lastly, music and movement therapies may enhance communication skills in children with other developmental disorders including children with dyslexia (Overy, 2003) and intellectual disabilities (Duffy and Fuller, 2000). Similar to children with autism, children with dyslexia have impairments in reading, phonological processing, and receptive vocabulary (Overy, 2000). Children with dyslexia significantly improved their spelling and phonological skills following a 15-week rhythm-based intervention involving singing and percussion games when compared to a control group receiving individual reading lessons (Overy, 2003). Engaging in timed rhythmic movement during singing may enhance the ability to parse words and give meaning to them during reading and verbalization (Sparks et al., 1974; Carroll, 1996; Overy, 2003, 2008; Roper, 2003; Overy and Molnar-Szakacs, 2009; Wan et al., 2011). This indirect linkage between perceptuo-motor and communication systems is shown in Figure 1. Children with moderate intellectual disability also showed improvements in verbal communication skills following an 8-week music therapy program (Duffy and Fuller, 2000). Overall, there is considerable evidence from music education, special education, and music therapies supporting linkages between musical experiences and communication development in children with autism, typically developing children, and children with other diagnoses.

### Effect of musical experiences on social-emotional development and behavioral skills

Music-making or singing in dyadic or group settings create opportunities for developing social connections. Synchronous movements during rhythmic actions or music-making as well as unison singing creates a state of social cooperation, shared purpose, and a sense of togetherness which sparks a social connection between individuals, as highlighted in Figure 1 (Marsh et al., 2009; Overy and Molnar-Szakacs, 2009; Kirschner and Tomasello, 2010). Moreover, group musical environments provide opportunities for learning social skills such as imitation, turn taking/social reciprocity, joint attention, shared affect, and empathy (Overy and Molnar-Szakacs, 2009), which are impaired in individuals with ASDs. While engaging in musical games, children will begin by imitating and synchronizing the actions of a social partner; however, gradually they will develop an understanding of their partner's intentions and emotions (Overy and Molnar-Szakacs, 2009). Overy and Molnar-Szakacs suggest that group music-making and singing conveys the affective state, physical state, and intentions of the partner and fosters empathy and positive emotions (Overy and Molnar-Szakacs, 2009). This could be particularly important in children with ASDs given their difficulties in empathizing and understanding the intentions of others (Koelsch, 2009). Moreover, different emotions such as happiness, sadness, fear, and anger can be effectively communicated to the listener through musical elements such as tempo and sound level of music as well as intonation and pauses in voice (Katagiri, 2009). Children with autism recognize affective signals conveyed through music, in spite of difficulties in recognizing emotions conveyed through speech (Heaton et al., 1999). Hence, we believe that socially embedded music and movement contexts involving listening, singing, moving, verbalizing, and playing, provide great opportunities to foster social connections and facilitate emotional understanding in children with ASDs. Further, the non-intimidating yet engaging nature of musical experiences and their ability to induce positive emotions while improving compliance may contribute to the behavioral effects of music therapies including a reduction in the frequency of negative behaviors. Conversely, the positive behavioral effects of music might in turn lead to enhanced social-emotional skills following musical training (see Figure 1).

Music-based interventions have been used to enhance social skills such as eye contact, engagement, and spontaneous initiation of social interactions in children with ASDs (Wimpory et al., 1995; Reitman, 2005; Kern and Aldridge, 2006; Kern et al., 2007; Stephens, 2008; Kim et al., 2009) (see Table 1 for details). A 12-week intervention of improvisational music therapy led to significant increases in the frequency and duration of shared positive affect and joint attention with the therapist in the music group compared to the control group engaged in toy play (Kim et al., 2009). Similarly, a 7-month intervention involving different types of rhythmic movement games to music between a child with autism and his mother led to an increase in the frequency of eye contact episodes and spontaneous initiation of interactions by the child, post-intervention (Wimpory et al., 1995). Music has been used to promote emotional understanding in children with autism. Specifically, when children with autism were taught the four emotions of happiness, sadness, anger, and fear using verbal instructions or appropriate background music or specially composed songs, they improved their understanding of the selected emotions most in the background music condition (Katagiri, 2009). Further, music-based contexts have been used with success to reduce challenging behaviors such as self-injurious, aggressive, and stereotypical behaviors in children with autism (Wood, 1991; Gunter et al., 1993; Clauss, 1994; Orr et al., 1998; Brownell, 2002; Pasiali, 2004; Rapp, 2007; Devlin et al., 2008; Carnahan et al., 2009a,b; Lanovaz et al., 2009).

Studies in typically developing adults and children in the field of social psychology provide substantial evidence for how musical experiences facilitate the social and emotional development of individuals. Healthy adults and children tend to synchronize more with a human partner than with a recording or a drumming machine (Himberg, 2006; Kirschner and Tomasello, 2009). Joint rhythmic activities may intrinsically motivate adults and children to move in synchrony and engage in a cooperative effort (Tomasello and Carpenter, 2007). There is a developmental trajectory for joint action in that adult-adult pairs demonstrate greater interpersonal synchrony during drumming than child-child pairs suggesting that synchrony during joint action is a learned skill that improves over development (Kleinspehn-Ammerlahn et al., 2011) There is objective evidence for both adults and children to exhibit more cooperative and empathetic behaviors toward their social partner after engaging in a synchronized group musical experience (Anshel and Kipper, 1988; Wiltermuth and Heath, 2009; Kirschner and Tomasello, 2010). Adults who had previously engaged in synchronized singing or movement were more likely to be cooperative during a group economic game compared to those who had engaged in unsynchronized activities (Wiltermuth and Heath, 2009). Similarly, children who participated in an interactive musical game with adult partners were more likely to exhibit prosocial behaviors of helping and cooperating with their partners compared to a control group that engaged in a dyadic, non-musical, storytelling activity (Kirschner and Tomasello, 2010). The authors proposed that musical experiences may provide greater opportunities for fostering social connections than just verbal and non-verbal communication (Kirschner and Tomasello, 2010). Overall, there appears to be promising evidence for the potential use of socially embedded music and movement games to facilitate the social-emotional and behavioral skills in children with ASDs.

### Effect of musical experiences on the refinement of gross and fine motor skills

Whole body rhythmic actions such as clapping, marching, or walking to music provide significant opportunities to facilitate gross motor skills. Temporal patterning is inherently present in musical rhythms and an effort to synchronize arm and body movements to the rhythm of music could promote motor coordination in children. In addition, musical experiences that require fine motor skills of playing various musical instruments such as the piano, guitar, or drums have the potential to promote fine motor coordination and motor sequencing/praxis by providing numerous opportunities to practice, refine, and appropriately time finger, hand, and arm movements (Rodriguez-Fornells et al., 2012). It is also suggested that adding music through music-supported therapies can enhance patient motivation and compliance, provide opportunities for extensive practice, and offer continuous auditory feedback for online corrections (Schneider et al., 2007; Rodriguez-Fornells et al., 2012). Children with autism have significant impairments in gross motor skills such as bilateral motor coordination (Green et al., 2009; Fournier et al., 2010), balance (Minshew et al., 2004), and gait (Vilensky et al., 1981; Hallett et al., 1993) as well as significant fine motor delays (Provost et al., 2007; Downey and Rapport, 2012) that could be addressed using music and movement games targeted toward specific motor skills. As mentioned earlier, to the best of our knowledge there is no study that examined the effects of music and movement interventions on the gross and fine motor skills of children with ASDs. Hence, we will mainly draw upon evidence from typically developing children and individuals with other special needs to support the use of music and movement games in promoting motor skills in children with ASDs.

Several music education approaches including the Dalcroze and Kodaly methods of musical learning promote gross motor performance (Findlay, 1971; Hurwitz et al., 1975; Bachmann, 1991; Frego et al., 2004). These approaches promote the use of body movements to understand musical rhythms, but in the process facilitate gross motor coordination and movement timing (Findlay, 1971; Hurwitz et al., 1975; Bachmann, 1991; Frego et al., 2004). There is some evidence for the use of these approaches to improve gross motor performance in typically developing children. Four to six-year-old typically developing children who received a 2-month music and movement program showed significant improvements in their gross motor skills such as jumping and dynamic balance as measured by the Motor proficiency test (MOT 4–6) compared to children who engaged in a non-musical, physical education program (Zachopoulou et al., 2004). In another comparative study, 4 to 6-year-old typically developing children who received a 10-week, Dalcroze-based integrated music and physical education program outperformed children who received a general movement exploration program on various custom-developed, perceptuo-motor skills, and creative movement activities (Brown, 1981). These studies suggest that rhythmic accompaniment during motor practice enhances gross motor skill learning in typically developing children. In terms of fine motor skills, typically developing children who received 2 years of piano instruction showed significant improvements in fine motor skills as measured by the response speed, visuo-motor control, and upper limb speed and dexterity subtests of the Bruininks Osteresky Test of Motor Proficiency (BOTMP) compared to children who did not receive piano instruction (Costa-Giomi, 2005). The fine motor improvements observed in the children were directly related to the duration of musical training (Forgeard, 2008). Overall, there is considerable evidence from the field of early childhood music education to support the use of music and movement games for gross and fine motor development.

There is some evidence from special populations including children with dyslexia and adults with Parkinson's disease (PD) supporting the benefits of rhythmic movement and dance-based interventions. Specifically, rhythm training involving whole body actions such as clapping and percussion games has been used to promote movement timing in children with dyslexia (Overy, 2008). Overy proposed that poor movement timing may contribute to the poor phonological awareness and reading deficits observed in children with dyslexia (Overy, 2003). Moreover, children with dyslexia were more inaccurate and variable during multi-limb motions such as walking and clapping to a metronome beat compared to typically developing children (Getchell et al., 2010). However, a short-term auditory pacing program improved the multi-limb coordination of children with dyslexia suggesting that auditory feedback might supplement existing kinesthetic and visual feedback, and thereby facilitate motor coordination (Getchell et al., 2010). Along the same lines, dance has been used to promote balance, gait, and functional mobility in adults with PD (Hackney et al., 2007a,b; Duncan and Earhart, 2012). Adults with PD have significant motor impairments including impairments of gait as well as static and dynamic balance, similar to the motor deficits of individuals with ASDs (Bloem et al., 2001). A 12-month, bi-weekly, community-based tango dance program in patients with PD led to improvements in balance, gait patterns, and movement control in the treatment group compared to the control group that received no intervention (Duncan and Earhart, 2012). Dancing involves practice of precise movement sequences that demand dual and multi-limb coordination with varying balance requirements, thus providing an excellent alternative treatment tool for individuals with movement impairments such as PD as well as autism (Earhart, 2009). In summary, there is evidence for the potential use of music-based movement experiences to promote gross motor and fine motor performance in typically developing children as well as in individuals with special needs. Given this evidence from music education and neurorehabilitation literature and the nature of the motor impairments encountered in autism, we strongly believe that it is important to systematically explore the effects of embodied music therapies on the fine and gross motor skills of children with ASDs.

### Musical experiences, perception-action linkages, and brain development

Multiple brain regions, including motor, perceptual, language, and social-emotional systems are stimulated during musical experiences due to their multimodal, multisystem nature. For example, while playing a musical instrument the musician reads the complex musical notation and translates it into highly time-locked, synchronized, sequential, and precise finger and hand movements. In addition, the musician will use the auditory feedback produced by his/her music to adjust the timing, spatial organization, and sequence of future movements (Zatorre et al., 2007). The very nature of this task demands a strong coupling between the auditory, visual, somatosensory, and motor cortices (Schlaug et al., 2010). In this section, we provide evidence for neural substrates that contribute to perceptuo-motor, communication, and social-emotional enhancement following musical training and their implications for individuals with autism.

Music produced during music making is a multimodal perceptual experience produced by the integration of sensory and motor systems involved in the experience (Phillips-Silver, 2009). During a musical activity, the movements produced by adults are intimately linked to the sounds perceived: what one hears depends on how one moves and vice-versa (Phillips-Silver and Trainor, 2007). Neuroanatomical evidence for a perception-action linkage during musical activities comes from brain imaging studies in trained musicians (Haueisen and Knösche, 2001; Gaser and Schlaug, 2003; Bangert et al., 2006; Habib and Besson, 2009). Musicians showed activity in the premotor areas while simply listening to piano melodies, whereas non-musicians did not show such activity (Haueisen and Knösche, 2001). However, non-musicians trained over 5 days to play a melody, demonstrated premotor cortical activity while simply listening to the trained melody; they did not demonstrate similar premotor activity on listening to an untrained melody suggesting the important role that perceptuo-motor mapping plays during the initial stages of learning (Lahav et al., 2007). Similar premotor activation is seen during both simple listening and covert/overt singing (Callan et al., 2006). Musical tasks involving only auditory, only visual, or only motor components led to co-activation of the auditory, visual, and motor areas suggestive of strong visuo-motor and audio-motor integration following musical training (Bangert et al., 2006). Similarly, presentation of musical notation alone led to co-activation in the visual and motor cortices following training in reading music and playing the keyboard (Stewart et al., 2003). Thus, there is considerable evidence for the ability of musical experiences to recruit multiple areas of the brain and promote multimodal integration.

The multimodal nature of musical experiences is especially important for individuals with autism due to their known deficits in multimodal integration (Minshew and Williams, 2007). According to the connectivity hypothesis, brains of individuals with autism are characterized by short-range over-connectivity and long-range under-connectivity (Belmonte et al., 2004; Courchesne et al., 2007). To be clear, there is an increase in the short-range cortico-cortical connections and an underdevelopment of long-range connections between different brain regions including the frontal, temporal, parietal, and subcortical areas (Belmonte et al., 2004; Courchesne et al., 2007). The impaired functions of long-range networks are thought to underlie the social-emotional and communication impairments of autism (Courchesne et al., 2007). Based on the evidence presented earlier, the ability of music to recruit multiple brain areas simultaneously might help address some of the multimodal integration deficits in autism. As an example, there is some evidence for a reversal in the left-right asymmetry in the arcuate fasciculus of non-verbal children with autism (Wan et al., 2012). The arcuate fasciculus is a long-distance white-matter tract that connects temporo-parietal areas with the frontal areas of the brain and is important for audio-motor integration of speech and language skills (Hickok and Poeppel, 2004; Glaser and Rilling, 2008). In healthy individuals, there is a left-right asymmetry in this tract with greater volumes in the left hemisphere than in the right hemisphere; in children with autism this asymmetry is reversed (Herbert et al., 2002; De Fossé et al., 2004; Wan et al., 2012) and is thought to underlie some of the language deficits in this population (Wan et al., 2012). However, there is promising evidence suggesting that novel music and movement interventions such as Auditory Motor Mapping Technique (AMMT) focused on promoting multimodal integration may in fact recruit these dysfunctional networks in children with ASDs (Wan et al., 2012, see Table 2 and within music therapy approach section).

**Table 2:** **Music therapy approaches: critical elements, domains of development, targeted skills, and populations**.

**Music therapy approach**	**Type of music therapy**	**Critical elements**	**Domains of development**	**Targeted skills and populations**
Auditory motor mapping technique	Active	Listening Singing Music-making	Communication	Speech sounds and word approximations in non-verbal children with autism (Wan et al., 2011)
Melodic intonation therapy	Active	Singing Gross-motor tapping	Communication	Phonation and speech production in children with apraxia (Roper, 2003)
Rhythm therapy	Active	Singing Music-making Rhythmic actions like clapping	Social communication	Movement timing, phonologic skills, auditory processing, and spelling in children with dyslexia (Overy, 2003)
Improvisational music therapy	Active	Music-making	Social communication Emotional	Eye contact, turn taking, spontaneous joint attention, behavioral compliance, and positive affect in children with autism (Kim et al., 2008, 2009)
Sound therapies such as Auditory Integration Therapy, Tomatis Method, and Samonas Therapy	Passive	Listening to music that has been modified by filtering and modulation	Sensory Behavioral Communication	Sound sensitivity, behavioral compliance, listening and comprehension. Majority of the studies found non-significant results for these outcomes (Rimland and Edelson, 1995; Bettison, 1996; Zollweg, 1997; Edelson et al., 1999; Mudford et al., 2000; Corbett et al., 2008)

Music and language systems also share common neural substrates. Specifically, the Heschl's gyrus, planum temporale, secondary auditory cortex, and the corpus callosum are all involved in both music and language processing (Meyer et al., 2002). Musical training leads to structural changes in the planum temporale, primary and secondary auditory cortices, and the Heschl's gyrus, all of which are important for auditory processing (Wan and Schlaug, 2010). Further, the magnitude of these changes is greater in musicians who begin training early in life (Gaser and Schlaug, 2003). Six-year old children who received musical training for 15 months demonstrated structural changes in the precentral gyrus, the corpus callosum, and the Heschl's gyrus (Hyde et al., 2009). Similarly, 9–11 year old instrumentalists with 4 years of musical training showed larger gray matter volumes in the sensorimotor and occipital cortices as well as greater activation of the mirror neuron systems (MNS) during rhythm and melody discrimination tasks compared to non-instrumentalists (Schlaug et al., 2005). Hence, in typically developing individuals, neuroanatomical evidence suggests strong links between musical training and activation of substrates common to both music and language processing.

There is clear evidence for the relatively unimpaired musical skills despite significant language impairments in individuals with autism (Bonnel et al., 2003; Heaton, 2003). There is also mounting evidence for abnormalities in neural networks underlying speech in autism (Hesling et al., 2010; Lai et al., 2012; Wan et al., 2012). A comparison of neural systems sensitive to both speech and music in low-functioning children with autism and age-matched healthy controls using functional magnetic resonance imaging and diffusion tensor imaging revealed that the activation in the inferior frontal gyrus in children with autism was lower than in controls during speech stimulation but higher than controls during song stimulation. This study argues for the potential utility of music-based therapies in remediating the core language impairments in autism (Lai et al., 2012). Some mechanisms have been proposed to explain the positive effects of musical training on speech impairments in autism. For instance, the OPERA hypothesis proposes that speech-related impairments could benefit from musical training due to its following characteristics—(1) *O*verlap exists in the brain regions processing speech and music (Patel, 2003), (2) *P*recision of processing required during musical activities is more intense than that needed for speech processing, (3) *E*motions invoked by musical activities are strong and positive, (4) *R*epetition and practice are the integral elements of all musical experiences, and lastly, (5) Focused *A*ttention is required for accurate musical performance (Patel, 2011). Taken together, these factors associated with musical training can drive experience-dependent plasticity in speech processing in individuals with autism (Patel, 2011).

Socially synchronous movements and unison singing during group music activities evoke the MNS activity in the brain. MNS has been postulated as the neural basis for social abilities of shared attention, affect, and empathy (Molnar-Szakacs and Overy, 2006; Cattaneo and Rizzolatti, 2009). The MNS includes a group of neurons thought to be present in the inferior frontal cortex, inferior parietal lobule, and superior temporal sulcus of the human brain (Buccino et al., 2004; Cattaneo and Rizzolatti, 2009). These neurons are activated both during action production and during observation of actions performed by others (Buccino et al., 2004; Cattaneo and Rizzolatti, 2009; Rizzolatti et al., 2009). An additional subset of premotor “mirror” neurons have been postulated to possess audio-motor properties so that they are activated just by listening to someone else singing or making music (Molnar-Szakacs and Overy, 2006). This may allow students to learn not just by playing the instrument on their own but also by listening to the sounds and watching the movements produced by their teacher (Schlaug et al., 2005). The shared and temporally synchronous activation of the MNS in individuals involved in a group music-making experience provides a neural basis for the shared experiences and social connections within the group (Molnar-Szakacs and Overy, 2006). There is mounting evidence that individuals with autism have a dysfunctional MNS which might underlie some of the social-emotional and motor imitation deficits observed in this population (Williams et al., 2001; Dapretto et al., 2005; Wan et al., 2010a,b). Hence, music-based activities involving imitation and rhythmic synchronization within socially embedded contexts may engage the dysfunctional MNS of children with ASDs (Wan et al., 2010a,b). Taken together, the neuroanatomical evidence presented in this section suggests that music and movement activities within social contexts can serve as a powerful medium to induce a range of plastic changes in brain structure and connectivity in individuals with ASDs.

## Propositions for using musical experiences in children with autism

Having reviewed strong behavioral and neuroanatomical evidence in favor of music and movement therapies for children with ASDs, we will now discuss the critical elements of musical experiences and their potential benefits for remediating the core impairments and comorbidities in autism. We will also review in detail the critical elements and potential benefits of three active music-based therapies that are currently utilized in the treatment of children with special needs.

### Critical elements of musical experiences for children with autism

Musical experiences can vary depending on the activities involved, but the four most critical elements are listening, singing, music-making, and rhythmic actions synchronized to music, experienced in individual or socially embedded, dyadic, and group activities (Edelson et al., 1999; Pellitteri, 2000; Schlaug et al., 2005; Overy, 2008; Wan et al., 2010a,b, 2011). Listening to music is predominantly a passive musical experience whereas singing, music-making, and rhythmic actions require active participation (Pellitteri, 2000). Each type of musical experience has its own applications. For example, passive listening techniques such as Auditory Integration Therapy (AIT) have been used to address behavioral problems and auditory hypersensitivity in children with ASDs (Rimland and Edelson, 1995; Bettison, 1996; Zollweg, 1997; Edelson et al., 1999; Mudford et al., 2000; Corbett et al., 2008); however, there is limited evidence to support their use (Sinha et al., 2011). Singing has been used as a communicative medium to compensate for language impairments as well as to promote language in individuals with various speech disorders including ASDs (Wan et al., 2010a,b). Music-making has been used extensively in music education to teach children concepts of rhythm, melody, and pitch as well as various spatio-temporal concepts such as slow-fast, soft-loud, moving on a count, etc. (Pellitteri, 2000). Specifically, improvisational music-making is an outlet for expression of creativity and individuality (Pellitteri, 2000). The last element of synchronized whole body rhythmic actions is often used to teach and internalize musical concepts such as rhythm. By grounding music in physical movements, eurhythmics provides an embodied musical experience (Findlay, 1971; Hurwitz et al., 1975; Bachmann, 1991; Frego et al., 2004). Structured and improvisational music-making as well as rhythmic whole body movements involve perception and action and promote fine and gross motor skills and bilateral and visuomotor coordination as discussed in the previous section (Phillips-Silver, 2009). Children can experience all the critical elements of music in individual as well as group settings. Individual experiences involve one-on-one interactions between the trainer and the child and are tailored to the individual needs of the child. Group sessions involve synchronous activities between members to ensure a meaningful and enjoyable musical experience and in turn facilitate social connections between members of the group (Pellitteri, 2000; Overy and Molnar-Szakacs, 2009). Moreover, careful additions of socially embedded, dyadic, and group activities would be important for children with ASDs to practice social communication skills.

### Current music therapy approaches used in children with autism and those with other special needs

Current music therapy approaches, their critical elements, domains of development, and targeted skills are highlighted in Table 2. In general, music therapies have been provided to children with ASDs (see Table 1 for details), dyslexia (Overy, 2003), apraxia (Roper, 2003), and intellectual disabilities (Duffy and Fuller, 2000) (see Table 2 for details).

1. *Auditory Motor Mapping Training (AMMT) and Melodic Intonation Therapy (MIT)* facilitate language production in non-verbal/low-verbal children by training an association between self-produced sounds (drum hit or finger tap) and articulatory movements or auditory-motor mapping (Sparks et al., 1974; Carroll, 1996; Roper, 2003; Norton et al., 2009; Wan et al., 2011) (see Table 2). AMMT combines critical elements of listening to the therapist's intonation and drum tapping, singing with the same intonation, and music-making by tapping on a pair of tuned drums. Therapists progress from sounding words and tapping the tuned drums alone to unison singing and music-making. It is proposed that ultimately the child produces the words on his/her own without any support from the therapist (Wan et al., 2011). Non-verbal children with ASDs demonstrated improvements in their ability to articulate words and phrases following an 8-week intervention of AMMT (Wan et al., 2011). Similarly, MIT which involves singing and associated gross motor tapping to mark the rhythm and stress of the intoned phrases was found to enhance phonation and speech production in children with apraxia (Roper, 2003; Norton et al., 2009).
2. *Rhythm training* has been used to address the timing deficits in language, motor control, perception, and cognition encountered in children with dyslexia (Overy, 2008) (see Table 2). Children with dyslexia significantly improved their phonological and spelling skills following a 15-week rhythm therapy intervention based on the critical elements of singing, joint music-making, and whole body rhythmic movements (Overy, 2008). The multisensory experience focused on rhythm and timing facilitated the temporal processing skills of children with dyslexia.
3. *Improvisational music therapy* is an individualized, patient-centered approach to facilitate social engagement and verbal and non-verbal communication skills in children with ASDs (Kim et al., 2009) (see Table 2). In this approach, the therapist uses improvised, shared music-making experiences to tune in to the patient's musical and non-musical non-verbal behaviors. Such moment-by-moment musical attunement of the therapist to the patient helps develop a medium of communication between the two, which in turn facilitates social skills such as turn taking, imitation, and joint attention as well as verbal communication skills (Kim et al., 2008). This approach has been used to improve social communication skills in children with autism (Kim et al., 2008, 2009). Taken together, several attempts have been made to therapeutically utilize the various critical elements of musical experiences in the treatment of children with autism and other pediatric disorders.

## Recommendations for clinicians and clinical researchers

In the above sections, we have reviewed vast evidence supporting the therapeutic use of embodied music interventions in addressing the multisystem impairments of children with autism and other similar developmental disorders. However, as outlined in the introduction, current research in this area has several limitations. In this section, we will provide recommendations for assessment and treatment of autism for clinicians and researchers working in this field. We hope that this discussion will provide guidelines for future systematic research on embodied music therapies and will bring multisystem music and movement interventions to the forefront in the treatment of autism.

### Recommendations for assessment of children with ASDs

In this review, we have offered substantial evidence on how musical experiences may impact the various forms of development in typically developing children and children with special needs. The majority of the evidence stems from literature in music education and special education and to some extent from the music therapy literature. Currently, there is limited evidence to support the use of music therapies in children with ASDs. Future research should consider using better study designs such as randomized controlled trials to examine the efficacy of music therapies on the various core deficits and comorbidities of children with ASDs. Standardized, reliable, and valid assessments should be routinely used to evaluate outcomes. In this section, we provide researchers with certain objective and subjective tools to better characterize their study populations and to assess the impact of music-based interventions on perceptuo-motor, communication, and social-emotional development. We strongly urge that whenever possible, researchers use a combination of subjective and objective tools to assess treatment effects.

To the best of our knowledge, no study to date has assessed the impact of music therapy on motor skills in ASDs. However, for future studies, we recommend that researchers consider the use of standardized tests such as the Bruininks Osteretsky Test of Motor Proficiency (BOTMP) (Bruininks, 1978), Sensory Integration and Praxis Tests (SIPT) (Ayres, 1996), Movement Assessment Battery for Children (MABC) (Henderson and Sugden, 1992), gross motor and fine motor subtests of the Mullen Scales of Early Learning (MSEL) (Mullen, 1995), and the Individualized Music Therapy Assessment Protocol (IMTAP) (Baxter, 2007) to assess for changes in motor function. In addition, context-specific changes in motor skills such as the accuracy of imitation or amount of time spent in synchrony can be examined using moment-to-moment video coding or quantitative measures such as relative phase analysis (Scholz and Kelso, 1989; Schmidt et al., 1991). Changes in sensory modulation could also be assessed using the Short Sensory Profile (Tomchek and Dunn, 2007), the sensory subtests of the IMTAP (Baxter, 2007), and the SIPT (Ayres, 1996).

Some common social communication measures for school-age children include the Assessment of Basic Language and Learning Skills-Revised (ABLLS-R) (Partington and Sundberg, 1998) and the Peabody Picture Vocabulary test (PPVT) (Dunn and Dunn, 1981). Non-verbal communication can be examined using the Early Social Communication Scale (ESCS) in young children (Mundy et al., 2003). In addition, researchers should also use video coding to measure socially directed verbal communication such as the frequency of spoken syllables/words, non-verbal communication such as social gaze, joint attention, and use of signs or gestures, as well as affective changes including durations or frequencies of positive, neutral, and negative affect.

For the assessment of changes in behavioral problems following intervention, several psychiatric measures, and parent/teacher questionnaires have been used. Some of the commonly used measures include the Brief Psychiatric Rating Scale (BPRS) (Lukoff et al., 1986), Repetitive Behaviors Scale-Revised (RBS-R) (Lam, 2004), Autism Behavior Checklist (ABC) (Krug et al., 1988), Pervasive Developmental Disorder Behavior Inventory (PDDBI) (Cohen and Sudhalter, 2005), Connor's Rating Scales (Conners, 1989), and the Aberrant Behavior Checklist (Aman and Singh, 1986). In addition, we recommend that researchers code for changes in the frequency of positive and negative behaviors within the music therapy sessions.

It would be important to characterize the study population given the diversity of impairments observed in ASDs. Group characterization measures include a confirmation of ASD diagnosis and its severity as well as a basic IQ measure. Autism-related impairments could be confirmed through medical records, screeners such as the Social Communication Questionnaire (Berument et al., 1999) or the Social Responsiveness Scale (Constantino and Gruber, 2002) or through gold-standard assessments/interviews such as the Autism Diagnostic Observation Schedule (ADOS-2) (Lord et al., 2012a,b) and Autism Diagnostic Interview -Revised (ADI-R) (Lord et al., 1994). Autism severity can be determined through standardized tests such as the Childhood Autism Rating Scale (CARS) (Schopler et al., 1980). IQ could be measured using various measures such as the Kaufman Brief Intelligence Test (KBIT) (Kaufman, 1990), Wechsler Intelligence Scale (WISC) (Wechsler, 1949), Stanford-Binet Intelligence Test (SBIT), (Terman and Merrill, 1960), or Differential Abilities Scale (DAS) (Elliott, 1990). Given the evidence for the multisystem effects of music interventions discussed, we urge researchers to assess the multisystem effects of music-based therapies using various sensori-motor, communication, social-emotional, and behavioral measures.

### Recommendations for treatment of children with ASDs

There is a strong need to further develop comprehensive, multisystem, music interventions to facilitate the communication, social-emotional, behavioral, and perceptuo-motor skills of individuals with ASDs. In addition, we have various specific recommendations on the nature, intensity, and frequency of music interventions. First, active music interventions that emphasize participation through singing, music-making, and synchronized rhythmic actions must be promoted as opposed to passive listening. Second, given the positive effects of socially embedded activities it would be useful to consider dyadic, triadic, or group-based activities. However, we acknowledge that working with children with autism is very challenging and the needs of each child are unique. The other members in the group could be typically developing siblings, parents, or caregivers who will adjust to the needs of the child. Third, we recommend better content development as opposed to purely improvisational music-based activities. Fourth, there is a need for better reporting standards while disseminating the results. Fifth, there is a need to test for skill generalization to novel contexts or standardized tests and maintenance of learned skills through long-term follow-up. Sixth, interventions should be offered within natural settings such as home or school environments to ensure ecological validity and generalization. In terms of the intensity of interventions, music-based interventions have been provided at least 2–3 times per week with each session lasting for ~30 min (see Table 1). Repetition is of utmost importance to ensure learning in this population. Hence, we recommend involving parents and caregivers in the training activities to enhance skill learning, generalization, and maintenance. Some additional special considerations specific to training sessions and needs of children with ASDs are listed in Table 3. These considerations incorporate the ideas promoted by contemporary autism interventions such as ABA, PECS, and TEACHH. The recommendations provided in this section should be used as guidelines; however the training protocols will need to be tailored to the individual needs of the child. As mentioned earlier, various domains of development can be addressed through music-based activities; however, certain domains may require more training than others for an autistic child due to his or her individual impairments. Similarly, specific modifications may be needed for a child due to his or her unique behavioral or sensory modulation impairments.

**Table 3:** **Special considerations for music-based interventions for children with ASDs**.

**Domain**	**Special considerations**
Structure of the environment	Predictability and familiarity is important. Follow a familiar activity schedule. Conduct sessions in the same physical space (Mesibov et al., 2004). Use visual cues to indicate the child's spot and distinguish the space used. Consider the needs of the child when setting up the environment. For example, avoid distractions, cover musical instruments until they are used, and avoid bright lights and loud sounds for hypersensitive children. Use visual picture schedules to provide structure to the session (Bondy and Frost, 2003). This helps children with ASDs to understand the progression in the session and helps them anticipate transitions. Allow time for the child to adapt to any new activity.
Instructions, prompts, and feedback	Be aware of the child's communication system in advance. Avoid long verbal instructions. Be brief and precise in your instructions. Whenever, possible, combine verbal and visual instructions. For example, use visual picture schedules and instructions such as “do this.” Make sure that the instructor is seated in front of the child to ensure that he/she is in the child's visual field. Instructions can be provided through songs to ensure better comprehension. A typically developing peer or adult could stand or be seated beside the child as a model for the child. One of the adults could provide manual guidance during the motor activities. Allow the child time to practice the activity independently (Shumway-Cook and Woollacott, 2007). Use props whenever necessary to clarify the goals of the activity.
Repetition and reinforcement	Repetition is the key for learning (Lovaas, 1987). Ask parents and caregivers to try out the activity in another environment to promote practice and generalization to other individuals and environments. Various rewards such as stickers and small toys could also be provided (Lovaas, 1987; Landa, 2007). Provide verbal and gestural reinforcement in the form of good jobs and hi-fives. Provide breaks from activity to do favorite sensory activities. Edibles should be used as the last resort.
Nature of the interaction	During group sessions, be sensitive to the individual needs of the child. Give sufficient breaks and avoid overwhelming the child. Try to keep the child actively involved as much as possible. Vary the level of task complexity. Use a mix of simple and complex activities to allow for success and engagement (Darrow, 2009). Within activities, vary the verbal and motor complexity. Allow time for free music-making and movements to sustain engagement. Look out for negative behaviors such as tantrums, non-compliance, and self-injurious behaviors. If these are observed, then ask the child to communicate that the activity be stopped. Seek advice from caregivers on best ways to address negative behaviors (Lovaas, 1987; Landa, 2007).

## Conclusions

In this review, we offered substantial evidence for the multisystem effects of musical experiences in children with ASDs, healthy individuals, as well as other pediatric neurological populations. We believe that novel, embodied rhythm-based, multisystem interventions grounded in singing, music-making, joint action, and social synchrony can be used to alleviate the core social communication deficits and perceptuo-motor and behavioral comorbidities of children with ASDs. Current evidence for the efficacy of music therapies in children with ASDs comes from a handful of studies that lack systematic study designs, assessments, and treatment protocols. There is an urgent need for systematic research in this field. Our research team has developed an intense, 8-week, novel, embodied musical intervention that will be tested within a pilot, randomized controlled trial to assess its effects on the multisystem performance of children with ASDs. If our hypotheses are upheld, we will be providing objective evidence to support the use of rhythm-based, music and movement intervention for children with ASDs. Future research should extend this work by examining multisystem effects of music therapies through larger clinical trials using larger sample sizes.

### Conflict of interest statement

The authors declare that the research was conducted in the absence of any commercial or financial relationships that could be construed as a potential conflict of interest.
